# A Rare Case of Hemodialysis as an Extracorporeal Treatment for Severe Phenytoin Overdose

**DOI:** 10.7759/cureus.70338

**Published:** 2024-09-27

**Authors:** Randy Felber, Victoria R Prager, Deena Werde, Bryan Dawkins, Ishan A Gunawardene

**Affiliations:** 1 Department of Foundational Sciences, Nova Southeastern University Dr. Kiran C. Patel College of Osteopathic Medicine, Clearwater, USA; 2 Department of Foundational Sciences, Nova Southeastern University Dr. Kiran C. Patel College of Osteopathic Medicine, Fort Lauderdale, USA; 3 Family Medicine, Lakeside Medical Center, Belle Glade, USA

**Keywords:** anticonvulsant drug, extracorporeal treatment, hemodialysis, intermittent hemodialysis, phenytoin, phenytoin overdose

## Abstract

Phenytoin is a well-known anticonvulsant medication that is useful in the management of most seizure disorders. Given the narrow therapeutic index of 10-20 mg/mL, acute phenytoin overdose can occur with either oral or intravenous administration. There is no distinct antidote to phenytoin, and therefore supportive care is the treatment of choice. Various methods to enhance the elimination have been discussed controversially and have shown a limited effect. We discuss a case in which intermittent hemodialysis was shown to significantly enhance the elimination of phenytoin in an acute overdose and drastically improve the clinical outcome. A 46-year-old male presented to the emergency department after a fall in which he injured his head and left knee. The patient admitted to ingesting 60 tablets of 100 mg extended-release phenytoin directly preceding the fall. He presented lethargic with slurred speech and ataxia. The patient was admitted to the ICU for observation with an initial phenytoin level of 50.1 mg/L. Phenytoin levels peaked at 68.7 mg/L on day two and continued to fluctuate. On day seven of admission, the level remained high at 62.6 mg/L, and at this point, the hospitalist, nephrologist, and poison control agreed to proceed with a trial of intermittent hemodialysis. After a four-hour dialysis session, phenytoin levels declined 27% to 45.6 mg/L and continued to fall. The patient's symptoms of slurred speech, drowsiness, and agitation remained throughout the hospital course but improved each day after hemodialysis. Phenytoin levels reached a therapeutic level of 19.5 mg/L on day 14 of admission, and the patient was alert, talkative, and asymptomatic. Intermittent hemodialysis was shown to be an effective elimination method in those with severe phenytoin toxicity and should be considered as a therapeutic option. Even with significant albumin binding, a single session of hemodialysis lowered the phenytoin levels by 27%. We also suspect the removal of additional toxins, and the desaturation of hepatic metabolism could have aided in the patient’s clinical improvement, further supporting therapeutic hemodialysis.

## Introduction

Phenytoin is a well-known anticonvulsant medication that is useful in the management of most seizure disorders. It works by blocking voltage-dependent sodium channels, limiting the propagation of seizure discharges. Given that phenytoin has a narrow therapeutic index, acute overdoses can result from overconsumption or excessive intravenous phenytoin administration in a hospital setting. The total serum therapeutic range is 10-20 mg/L. With levels greater than 20 mg/L, toxicity can occur resulting in a spectrum of symptoms including ataxia, slurred speech, lethargy, seizures, coma, and even death. Upon presentation of a patient with phenytoin intoxication, the treatment is typically the same despite the phenytoin level. There is no distinct antidote to phenytoin, and therefore supportive care is the treatment of choice. This includes correcting any hemodynamic instability, creating an advanced airway if impeding respiratory depression, and correcting any electrolyte imbalances [1]. Poison Control commonly recommends activated charcoal, a commonly used gastrointestinal decontaminant, if given soon after known ingestion and is most effective if given within one hour.

Recommendations on further treatment to enhance elimination through extracorporeal methods have been under debate with only a few successful cases reported. Extracorporeal elimination is typically done using hemodialysis, hemofiltration, plasma exchange, and other similar techniques that work to filter out and remove the drug from the patient's circulation. Due to phenytoin being almost completely albumin-bound, hemodialysis would theoretically be of limited benefit. We discuss a case in which one episode of intermittent hemodialysis was shown to significantly enhance the elimination of phenytoin in an acute overdose and drastically improve clinical outcomes.

## Case presentation

A 46-year-old male with a past medical history of seizures presented to the emergency department covered in urine with an altered mental status and a laceration to the left side of his forehead. The patient was found on the ground after falling on his left side and hitting his head and left knee. At that time, he was following basic commands, but he was staggering and had slurred speech. The patient was previously given a 90-day supply of extended-release 100 mg phenytoin pills for his seizures by a primary care doctor but only a 30-day supply remained after the incident. The medications were packaged in blister packages with 30 pills in each package. The patient admitted to taking approximately 60 pills the day he presented to the emergency department.

In the emergency department, the patient was drowsy and sluggish and had to be awakened repeatedly to complete intake questions. He was able to correctly state his name, date of birth, and the current year. The patient was lethargic with slurred speech but had no focal neurological deficits. A head computerized tomography (CT) was negative for any acute changes. Phenytoin levels were 50.1 mg/L (therapeutic levels: 10-20 mg/mL) on admission. Poison Control was contacted regarding recommendations and suggested continuing to monitor phenytoin levels, checking vitals, and performing neurologic checks. While Poison Control also recommended activated charcoal treatment, the Emergency Department attending physician decided to hold off on administering based on the time since ingestion was over three hours since consumption. The patient was admitted to the intensive care unit for further management.

Approximately 39 hours after the initial presentation, the phenytoin levels reached their peak of 68.7 mg/L. On this day, the patient became agitated, and while attempting to get out of bed, he fell due to being restrained to the bed by handcuffs that were in place due to the patient’s agitation and potential for violence toward hospital staff. The forehead laceration was reopened and had to be re-sutured. A second repeat CT of the head showed no acute changes. While a wound to the left knee was obtained during the incident, no acute fracture or dislocation was found. The phenytoin levels started to decline over the next two days reaching an initial low of 55.7 mg/L but then began to rise again.

On hospital day seven, the patient became agitated and pulled off the telemetry monitor and intravenous (IV) line and attempted to stand up from the bed but fell again due to the restraints. A third CT of the head and X-ray of the bilateral knees found no acute findings. A CT of the abdomen and pelvis to determine if there was any trauma from the fall showed dependent basal densities in the posterior aspects of both lower lobes. No free air, inflammatory changes, abdominal masses, adenopathy, or ascites were noted. The patient was also found to have a temperature of 38.5 degrees Celsius (normal range 36.5-37.5 degrees Celsius), and later in the day, the patient became altered, including unable to follow commands, increased severe slurring of speech, and with delayed response to questions. The patient then had an episode of emesis in which a nasogastric (NG) tube was placed. An abdominal/pelvic X-ray showed no appearance of a bowel obstruction. A magnetic resonance imaging (MRI) scan of the brain and a magnetic resonance angiography (MRA) of the head and neck revealed no acute changes to the vasculature or soft tissues and ruled out further acute changes. Serum ammonia was also found to be mildly elevated but was quickly corrected with oral lactulose. The patient was also found to have a urinary tract infection with *Enterococcus* and was started on IV antibiotics.

**Figure 1:**
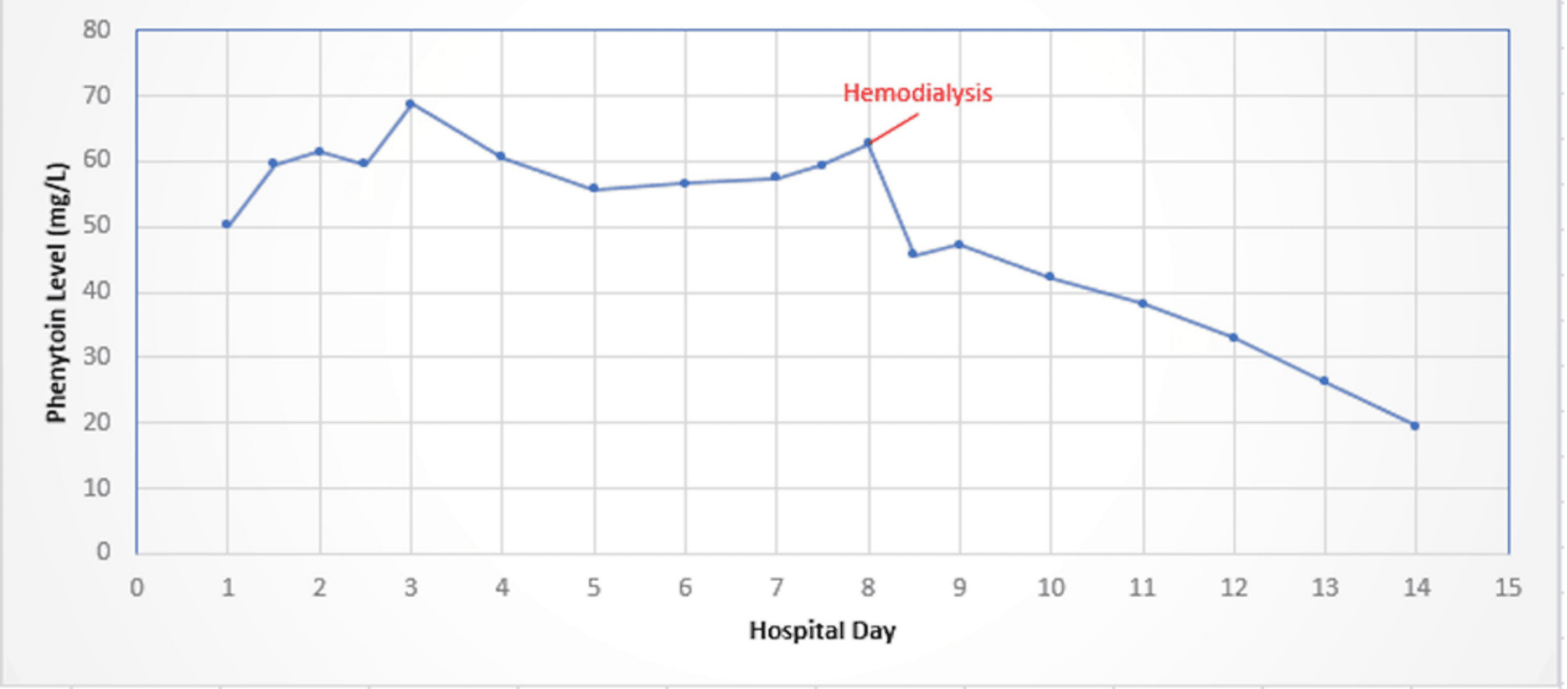
Serum phenytoin levels throughout the patient's time spent in the hospital.

On hospital day eight, dexmedetomidine was started by the intensivist in an effort to reduce the patient’s ongoing agitation and violence. Phenytoin levels reached a second peak of 62.6 mg/L, after which Poison Control was contacted along with nephrology to discuss the rise in levels eight days after consumption and the patient's rapidly declining state. Poison Control suggested the possibility of continued ingestion of phenytoin possibly being stashed rectally and recommended a polyethylene glycol bowel cleanse in response. The patient had been accompanied by an assigned sitter and security guards at the bedside due to the patient’s violence since admission, so the possibility of rectal stashing of phenytoin pills was ruled out. Using dialysis for hemofiltration was also discussed at this time, and the decision to proceed with intermittent hemodialysis was made. A right femoral Quinton catheter was placed, and a four-hour hemodialysis session was performed. Blood flow rate was maintained at 200 mL/min with a dialysate flow rate of 600 mL/min. The ultrafiltration rate during the treatment was 130 L/min across a semipermeable membrane with a transmembrane pressure of 40 mmHg. Normal ranges for these flow rates vary based on the purpose of the hemodialysis and the weight of the patient. These rates were chosen by the dialysis technician based on the hospital protocol for intermittent hemodialysis. Approximately one hour after hemodialysis, the phenytoin level declined 27% to 45.6 mg/L and continued a downward trend. Given the reduced agitation, aggressiveness, and phenytoin levels, dexmedetomidine was weaned off within the next 24 hours. The figure below illustrates the serum phenytoin level over time and highlights the drastic decline following hemodialysis (Figure 1).

Two days later, on hospital day 10, the phenytoin level was down to 42.2 mg/L. The patient had another episode of agitation, which included screaming, attempting to remove his mittens, wiping feces on the bed, and spitting at a staff member. Haloperidol and lorazepam were administered at this time, and the dexmedetomidine was restarted. Over the course of the next two days, the phenytoin level continued to decline, and the patient started answering questions appropriately, remained alert, and was able to be weaned off dexmedetomidine again.

On hospital day 14, the phenytoin level reached therapeutic range with a level of 19.5 mg/L. When the patient was seen at the bedside, he was sitting up in bed, smiling, talkative, apologetic, and answered questions with ease. The patient no longer showed any clinical signs of phenytoin toxicity and was cleared for discharge. The patient admitted that the overdose of phenytoin was an intentional suicide attempt. His prescription for phenytoin was switched to levetiracetam 500 mg twice daily, an anticonvulsant with a wider therapeutic index and a lower side-effect profile. Sutures from his forehead laceration were removed, and the patient was discharged.

## Discussion

Phenytoin is a well-known anticonvulsant medication that is useful in the management of most seizure disorders. It blocks voltage-dependent sodium channels, limiting the propagation of seizure discharges. Given that phenytoin has a narrow therapeutic index, acute overdoses can result from excessive intravenous phenytoin or fosphenytoin administration in a hospital setting or from drug overconsumption. The total serum therapeutic range is 10-20 mg/L. Phenytoin binds extensively (90%) to serum proteins, especially albumin, and only the free drug is pharmacologically active. Phenytoin is metabolized by hepatic microsomal enzymes into inactive metabolites. The free and conjugated forms are then eliminated in the urine after undergoing enterohepatic recycling. In therapeutic doses, the peak phenytoin level occurs between 90 minutes and three hours if taken orally. The mean plasma half-life is approximately 22 hours, but this varies from patient to patient. Differences in the rate of metabolism between individuals can be due to genetic variability in phenytoin uptake and in cytochrome P450 enzymes (CYP2C9). In the setting of acute ingestions, ongoing absorption can last weeks; this might be due to phenytoin’s low water solubility, its effect on reducing gastrointestinal motility, or the hepatic clearance being saturated, which shifts the elimination from first- to zero-order kinetics, therefore slowing the elimination [1].

Phenytoin levels are typically greater in the central nervous system than in the circulation. The clinical presentation may vary greatly depending on the plasma concentration and from case to case. Seizures are not commonly seen in phenytoin poisoning but may occur with concentrations greater than 50 mg/L, coingested medications, trauma, drug or alcohol withdrawal, and infection. Cardiovascular effects such as hemodynamic instability and arrhythmias can arise with intravenous administration. With concentrations of more than 120 mg/L, death can occur [1]. The most common signs and symptoms relating to the serum phenytoin concentrations are described in Table 1.

**Table 1:** Clinical signs and symptoms that correlate with the serum phenytoin level.

Phenytoin level (mg/L)	Clinical presentation
<10	Usually no effects
10-20	Therapeutic range
20-30	Spontaneous nystagmus
30-40	Vertical nystagmus, diplopia, ataxia, slurred speech, tremor, hyperreflexia, nausea, and vomiting
40-50	Lethargy, confusion, disorientation, hyperactivity, other movement disorders
>50	Coma, seizures

There is no distinct antidote to phenytoin, and therefore supportive care is the treatment of choice. This includes maintaining critical functions, monitoring a conscious state, avoiding fall-related injuries, and managing nausea, vomiting, and possible seizures. Dehydration can result from the nausea and vomiting brought on by phenytoin overdose; thus, antiemetics and intravenous fluid replacement should be used as treatments. Electrolyte disturbances and hypoglycemia may also occur and should be treated if present. In the instance of seizures occurring during phenytoin overdose, benzodiazepines and phenobarbital may be used according to treatment guidelines. Endotracheal intubation should be performed if there is a persistent decreased conscious state risking airway compromise. Various methods may be used to reduce phenytoin’s absorption, but it is debated whether these actually change clinical outcomes. For example, gastric emptying should not be considered a routine treatment for phenytoin toxicity. Activated charcoal effectively binds a variety of drugs, including phenytoin, and is often recommended only within the first hour or two after drug ingestion due to its effects rapidly diminishing with time [1]. However, the literature, such as an article published in the American Journal of Clinical Toxicology, highlights important points that raise a debate on whether it impacts clinical outcomes [2].

Due to phenytoin being close to 90% albumin-bound, traditional hemodialysis will have limited effect in lowering levels. Patients who have known kidney failure may have a worse presentation as the drug may be displaced from albumin by uremic toxins, which can induce intoxication from increased free phenytoin levels. Removal of these uremic toxins, by using dialysis with a high-flux membrane, can theoretically reduce free phenytoin levels [1].

In 2016, the Extracorporeal Treatments in Poisoning (EXTRIP) Workgroup performed a thorough review of current literature to create evidence-based guidelines on the use of extracorporeal treatment (ECTR) in patients with phenytoin intoxication. Based on 31 patients in 30 different case reports, the workgroup concluded that phenytoin is moderately dialyzable despite its high protein binding. In certain severe cases of phenytoin overdose, where a protracted coma or profound ataxia is anticipated, the use of ECTR could be justified. Intermittent hemodialysis (HD) is the recommended ECTR modality for phenytoin intoxication; however, if hemodialysis is not available, hemoperfusion is a suitable substitute [3].

The use of hemodialysis as the sole ECTR method to enhance the removal of phenytoin has rarely been used and was found to be effective in a few cases similar to ours. One of these involved a six-hour hemodialysis session that decreased the phenytoin levels by 47% and markedly improved the patient's level of consciousness [4]. Another case utilized an eight-hour HD treatment with a high cut-off filter, allowing the removal of molecules up to 45 kDa, which decreased the phenytoin level by 28.5%. The large surface area of the Theralite filter was determined to be the most beneficial aspect of the treatment [5]. The rarity of utilizing HD in phenytoin toxicity has further highlighted our case in which HD was successful.

Adding hemoperfusion to intermittent HD to absorb the phenytoin directly as it passes through a cartridge containing either charcoal or a resin has been discussed as possibly superior to other ECTR modalities. Very few cases have been published utilizing this method [6]. One case described an intravenous toxicity of up to 117 mg/L in which multiple rounds of hemodiaperfusion was used and considerably reduced the half-life of phenytoin to about seven to 13 hours compared to the native half-life ranging from 40 to 100 hours [7]. Hemoperfusion has therefore been reported to reduce plasma phenytoin concentration but can be counteracted by rapid redistribution of phenytoin from the gastrointestinal tract, which could result in a return to pre-hemoperfusion concentrations [8].

Molecular Adsorbent Recirculating System (MARS) has been discussed as a possible elimination method and was reportedly effective in the case of phenytoin toxicity. MARS uses hemofiltration along with an extracorporeal liver support device, which eliminates albumin-bound toxins based on the concept of albumin dialysis. This concept is demonstrated by the use of activated charcoal and anion-exchange resin, which filters out and washes the albumin before it returns to the patient and recirculates. In the context of acute renal failure and hemodynamic instability, the case described a patient who developed phenytoin intoxication as a result of IV administration. The patient showed a significant decrease in serum phenytoin levels after an 11.5-hour treatment, which was accompanied by a two-day improvement in clinical status. It was discovered that the activated charcoal filter was the primary method of phenytoin elimination in this patient. Despite the positive response in this case, MARS is not recommended given the lack of sufficient clinical research and evidence [9].

Urgent lowering of plasma phenytoin concentration may reduce the risk of cerebral toxicity, fall risk, and ataxia, shorten the length of stay in the ICU, and therefore be beneficial. The discussion of whether the risks involved in the use of ECTR elimination modalities outweigh possible benefits is still unanswered. Aside from the very few single case reports, there is still a lack of evidence supporting the routine use of enhanced elimination modalities. In our patient, the phenytoin plasma concentration was 62.6 mg/L before hemodialysis in contrast to 45.6 mg/L after hemodialysis. This 27% drop in plasma concentration and clinical improvement may be explained by the clearance of other toxins and free radicals during hemodialysis, the potentially lower half-life of phenytoin, or the desaturation of metabolism in the liver allowing for increased clearance. Caution should be taken when performing hemodialysis in phenytoin toxicity due to the potential for rebound toxicity.

## Conclusions

The patient described in this case had a severely toxic phenytoin level that persisted for over a week which resulted in clinical signs and symptoms of central nervous system toxicity. We illustrated a significant improvement in the plasma phenytoin level and clinical status through the use of intermittent hemodialysis. Even with significant albumin binding, phenytoin levels in our patient dropped by more than 27% with the use of a single session of hemodialysis. Hemodialysis was proven here to be an effective ECTR elimination method in severe phenytoin toxicity and should be considered a therapeutic option in the management of these patients.
